# Cytopathological Study of the Circulating Tumor Cells filtered from the Cancer Patients’ Blood using Hydrogel-based Cell Block Formation

**DOI:** 10.1038/s41598-018-33464-1

**Published:** 2018-10-12

**Authors:** Yoon-Tae Kang, Young Jun Kim, Tae Hee Lee, Young-Ho Cho, Hee Jin Chang, Hyun-Moo Lee

**Affiliations:** 1 Cell Bench Research Center, Korea Advanced Institute of Science and Technology, 291 Daehak-ro, Yuseong-gu, Daejeon 34141 Republic of Korea; 2 Research Institute and Hospital, National Cancer Center, 323323 Ilsan-ro, Ilsandong-gu, Goyang-si Gyeonggi-do 10408 Republic of Korea; 3 Samsung Medical Center, 81 Irwon-ro, Gangnam-gu, Seoul 06351 Republic of Korea; 4 Present Address: College of Engineering, University of Michigan, 2800 Plymouth Road, Ann Arbor, 48109-2800 United States; 5 Present Address: Department of Nanoengineering, University of California, San Diego, La Jolla, California 92093 United States

## Abstract

Circulating tumor cells have emerged as biomarkers for estimating the tumor burden and metastatic potential of cancer patients. However, to date, most of studies and applications of circulating tumor cells have been conducted and applied to epithelial cancers such as breast, colorectal, and prostate tumor. The only FDA-cleared method, CellSearch, makes use of antibody against epithelial surface protein expressed on CTCs, thus obstructing wide application for various cancers with non-epithelial and semi-epithelial characteristics including renal cell carcinoma. Due to rarity and ambiguity of CTCs, designed experiment including non-biased CTC isolation and subsequent cytopathological study for finding applicable immunomarkers are urgently needed for clinical use of CTCs for less-studied cancers. Here, in order to construct the fundamental step for CTC diagnosis without limitation of its epithelial characteristics, we present the simple and novel method which incorporate both label-free CTC isolation and pathological study using hydrogel-based cell block formation. Six cell lines from lung, ovarian, kidney cancers were used to make cell block and analyzed by conventional immunocytochemical staining method to find the candidate markers for CTC. Especially for renal cancer, the physically isolated CTCs were further immunocytochemically examined with the screened candidate markers by cell block construction, and verified their clinical utility using blood samples from patients with renal cell carcinoma. This comprehensive study demonstrates that the present approach can be used to find the potential markers for any type of cancers regardless of their epithelial characteristics and isolate the specific type of CTCs in label-free manners.

## Introduction

Circulating tumor cells (CTCs) is defined as tumor cells shed from the primary tumor site, circulating along the blood vessels, thus forming secondary tumor, which is called metastasis. The CTCs have been considered as one of the promising biomarkers to give information of current tumor status and metastatic potential. Recent works have showed that CTC number in blood is closely related to aggressiveness of tumor and change of number also reflects the susceptibility to anticancer drugs applied to patients with cancer^1^. Notwithstanding its significance and importance in cancer progression, CTC-based checkup has not been incorporated widely into clinical practice, such as evaluation of cancer progression and finding optimal anticancer drugs. Until now, the one and only FDA-cleared CTC diagnostic tool is CellSearch, but even this tool received its clinical availability in three cancers only, metastatic breast, prostate and colorectal cancer. The so-called ‘gold standard’ of CTC-based diagnostic tool, CellSearch, and its following CTC isolation techniques^2,3^ mostly rely on the antibody against epithelial cell adhesion molecule (EpCAM), which is normally expressed on epithelial cancer cells only. EpCAM is still widely used for CTC isolation and have been accepted as the CTC marker due to their ubiquitous expression on epithelial CTCs, albeit at variable levels. However, in some types of tumor cells, EpCAM expression is down-regulated and even in epithelial cancers, the expression level of EpCAM can be turned into weak- or negligible level after epithelial-mesenchymal transition (EMT), which is natural and inevitable pathway of tumor progression^4^. To overcome this limitation, label-free circulating tumor cell isolation methodologies^5–8^ have been studied and shown comparable or even higher detection sensitivity on certain cancers with the possibility on systematic study of CTCs^9,10^. In spite of remarkable number of alternative approach for CTC isolation, the method isolating CTCs universally in cancers and comparable for subsequent CTC study has not been developed yet.

Meanwhile, there are several attempts to study rare cells systematically, including circulating tumor cells. Single cell analysis (SCA) is recently accepted as the tool for studying cellular heterogeneity in protein, nucleic acids, and metabolites^11,12^, and has identified unknown cell types and associated markers. The fluorescence activated cell sorters (FACS), one of the SCA methods, had been applied to find the expression patterns in proteins on cells. In addition, recently this technique successfully captured single CTC, however, its inherent systematic losses of cells remained problematic. Also, this technique limited to multiple marker validation due to fluorescence overlapping^12,13^. The formalin fixed paraffin embedded (FFPE) tissue specimen is routinely used for clinical practice^14^. The inherent advantages on FFPE, such as including cost-effectiveness and convenience allow us to use it widely. Recent advance in image processing led FFPE tissue specimen to be used for multiplexed single-cell analysis^15^. However, FFPE specimen, originally developed for tissue study, is difficult to be incorporated for rare cell application. Therefore, additional efforts in rare cell block formation are needed.

Renal cell carcinoma (RCC), also known as renal cell adenocarcinoma, is the most common type of kidney cancer^16^, and shows a relatively better prognosis in early stage; but, 5-year survival rate is reduced considerably when the cancer has spread^17^. Although early diagnosis is important for the patients with RCC, approximately 25–30% of patients were diagnosed with metastasis^18,19^, due to silent clinical symptom and high tendency to invasion to renal vein. At the late stage, the survival rate of the patients has crucially diminished due to the low efficiency of systemic treatment and poor response of cytotoxic agents^20–22^; RCC is usually considered as the most chemo-resistant tumors^23^. The genetic analysis, which is actively studied for breast, colon, and lung cancer, has been limited to use in renal cell carcinoma. Unlike other cancers, the most common type of RCC, clear cell renal cell carcinoma (ccRCC) has shown less than 20 DNA copy-number alterations and fewer change^24^ with its innate significant mutation heterogeneity^25,26^.

Many researchers have been trying to find a novel biomarker, which indicate important clinical events such as cancer onset, recurrence, or progression^27^. There are few attempts to study CTCs in patients with RCC. In 2009, K. Bluemke et. al. identified CTCs in 96 (41%) of 233 peripheral blood samples, which originated from 81 (53%) of 154 RCC patients^28^. Meanwhile, A. Gradilone et. al. investigated 25 metastatic RCC (mRCC) patients using the CellSearch, and they found the presence of CTCs from 4/25 patients (16%)^29^. The limited detection ability as well as their biased specificity for RCC struggle from understanding the comprehensive characteristics of CTCs from RCC. In addition, because of their semi-epithelial characteristics, CTC isolation using CellSearch for RCC can be limited for RCC patients. Similar to RCC, other type of cancers, including lung and ovarian still have difficulty in capturing heterogeneous CTCs and finding clinical importance of CTCs captured by EpCAM-based methods.

Recently, our group reported two meaningful progresses in CTC researches: 1) label-free CTC isolation method for heterogeneous CTC^30^ study and 2) cell block^31^ method for finding applicable immunomarkers and cytopathological study. The CTC isolation and subsequent cytological study of the isolated CTCs were conducted in some studies^32–34^. The CTCs from patients with hepatocellular carcinoma isolated by track-etched filter were cytopathologically examined, and showed that those cells are anti-AFP positive^33^. Clustered CTCs isolated by filter-styled microfluidics device was assayed immunocytochemically and revealed that those cells in cluster express proliferation marker, Ki-67^34^. However, most of previous studies used immunofluorescent staining, which is limited in cytologic evaluation of cellular details, and yields false-positive (non-tumor cell) or false-negative (non-reactive tumor cell) results. In addition, cytopathological rare cell studies were also conducted as supplementary confirmation method, and even analyzed with a small number of well-known markers. Here, we present the systematic study for finding applicable markers using hydrogel-assisted hydrogel cell block, and its clinical application with CTCs isolated by label-free manner and stained cytopathologically. Immunocytochemical (ICC) stain has a merit of morphologic evaluation for cytological details, leading to detection of marker-negative CTCs (false negative by immunofluorescent stain) or marker-positive non–tumor cells (false positive by immunofluorescent stain).

**Figure 1:**
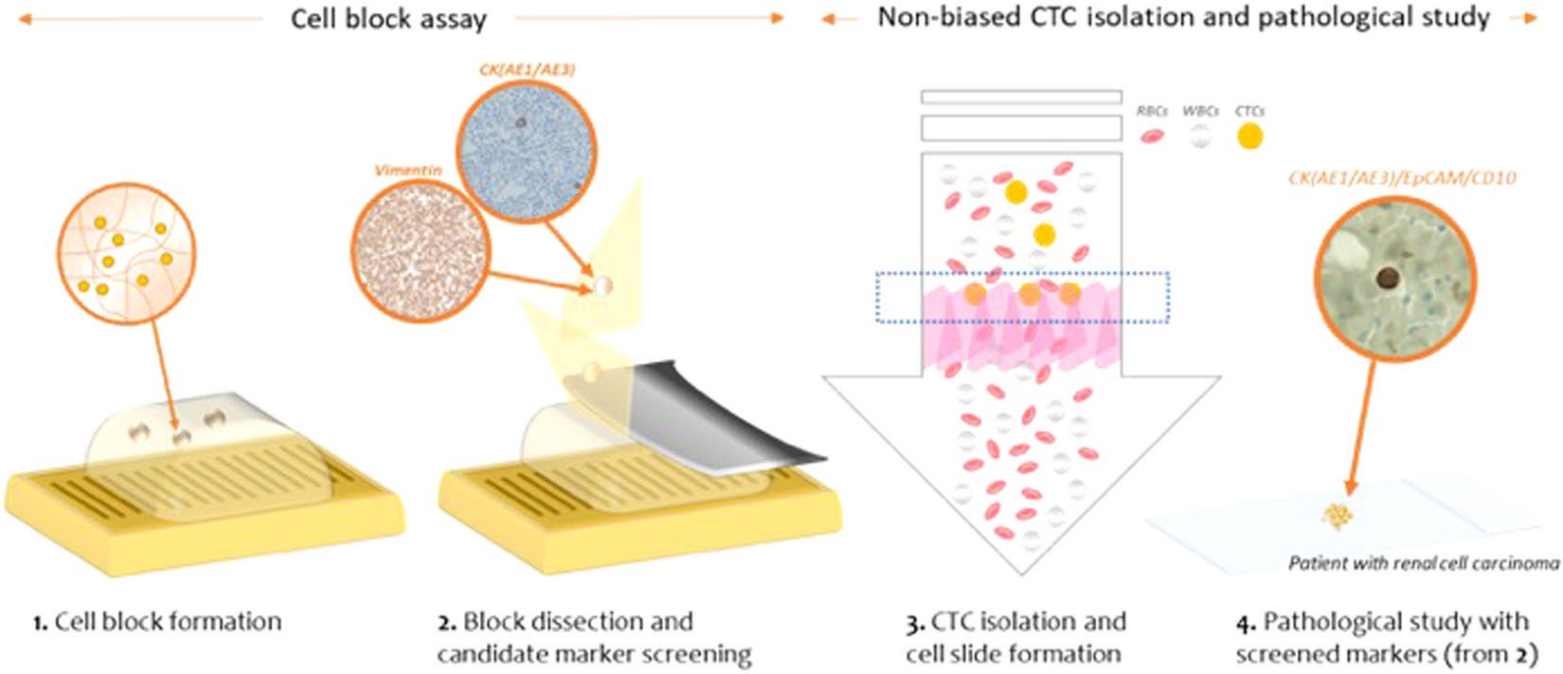
The schematic diagram of the present work for the pathological study of the circulating tumor cells filtered from the cancer patients’ blood samples.

First, we developed the methods which capture CTCs based on their size and deformability while maintaining their physical shape and viability, and showed that this method facilitate heterogeneous CTC subtypes isolation which have been limited by previous EpCAM-based CTC isolations. By help of harvesting heterogeneous subtypes of CTCs, various CTC studies including screening of applicable CTC markers can be possible. Second, we proposed the novel and versatile procedure to find the applicable CTC markers using formation of cell block containing CTCs and blood cells. Like paraffin-based tissue blocks, the present hydrogel-based cell block is compatible to conventional immunohistochemical (IHC) staining procedures. The fabricated cell block is serially sectioned into thin slices, transferred onto slides, stained with multiple markers, thus confirming the expression of multiple candidate markers which are available to specific cancers. In this study, we used two methods in order to construct the basis for cytopathological analysis of CTCs in cancers which have been rarely studied in terms of CTCs so far (Fig. 1). Using six cell lines from lung, ovarian, and RCC, we first formed hydrogel-based cell blocks for finding applicable immunomarkers for three cancers. Especially for RCC, the candidate markers, screened by cell block assay, were applied to cytopathological study of the isolated CTCs by our newly developed label-free CTC isolation microfilter from the 48 RCC patient blood samples. This comprehensive study will be helpful for both finding undiscovered immunomarkers of CTCs and studying their clinical roles without biased view of them.

## Results

### Cell block formation using cancer cell-laden hydrogel bead

**Figure 2:**
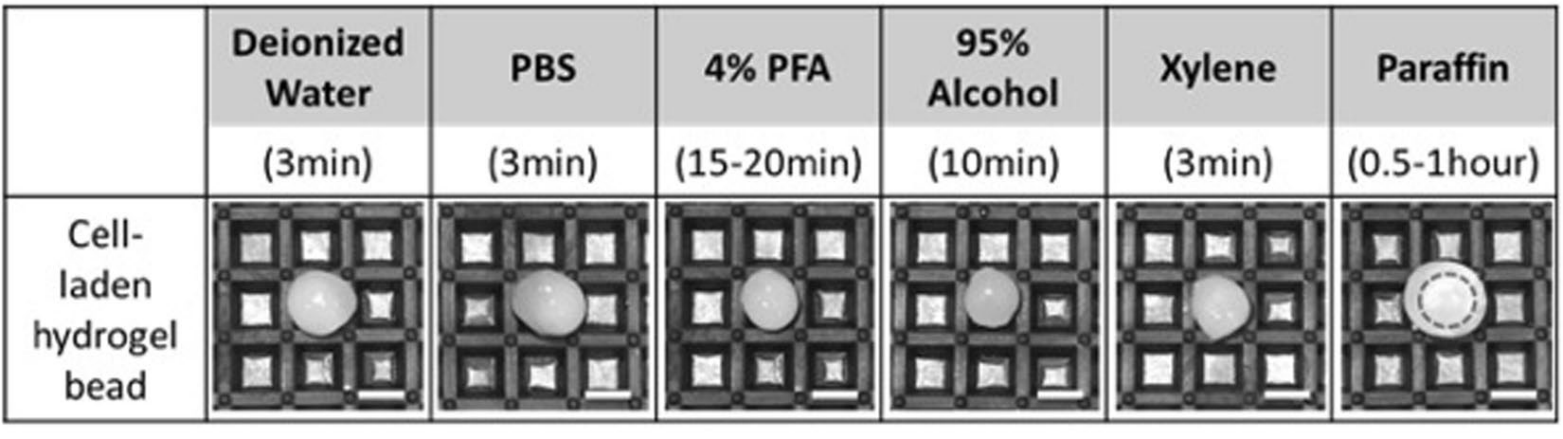
The change of cell-laden hydrogel bead for cell block formation after dipping in various solutions (Scale bar = 3 mm).

To test the durability of fabricated cell-laden hydrogel beads in various essential solutions for IHC, we first made beads using fluorescence labeled lung cancer cells, then dipped in and out the fabricated beads in 6 different solutions, deionized water, PBS, 4% paraformaldehyde, 95% ethanol, xylene, and paraffin. After dipping in for 3 to 60 minutes, we evaluated the morphological changes of hydrogel beads. The hydrogel beads after dipping in 6 different solutions are shown in Fig. 2. All tests were conducted independently using 6 different hydrogel beads, but their size of starting beads were identical. Considering its original deviation in size, each bead maintained its original shape. For paraffin, it might seem to become bigger than original one but it is because of solidified paraffin covering hydrogel bead after dipping out. Those beads were also examined by fluorescence imaging system to see the cells inside the hydrogel beads. In spite of deviations between beads under different solutions, all hydrogel beads show its fluorescent coming from the cell contained and it showed that the fabricated hydrogel bead are endured in various solutions for IHC and can be used for this study.

We also examined the change of hydrogel beads in serial procedures from fixation to formation using hydrogel beads containing 6 different cancer cells. Each cell-laden hydrogel bead remained intact after continuing dipping in and out from various solutions, such as 4% paraformaldehyde, 70–100% ethanol, xylene, and paraffin. Especially, in clearing process using xylene, the cell-laden hydrogel was turned into transparent, however, the bead still kept its original shape. After impregnation step, which is covering with paraffin wax, the overall volume of cell-laden hydrogel bead was noticeably diminished because of serial dehydration process and long-incubation time in paraffin wax (~1 hour). However, the final form of cell block did not matter for embedding, section cutting, and staining steps.

### Optimal staining protocol for cytopathological study of CTCs

Generally, protocols followed in histo-techniques include fixation, dehydration, clearing, impregnation, embedding, section cutting, staining, and mounting. In order to find the optimal protocol for cell-based pathological study using cell-laden hydrogel, first we tested the applicability of various solutions for fixation. Compared to other procedures for IHC, fixation has various options to choose depending on the sample used and staining lesion. In order to see the difference between them and find the optimum, three different fixative solutions, 95% ethanol, 100% methanol, and 4% paraformaldehyde were applied to slide containing ovarian cancer cells (OVCAR3) and lung cancer cells (A549). In order to see the effects of these solutions to hydrogel beads, we additionally made the hydrogel bead containing fluorescence labeled A549 then examined the morphology of bead and cells in hydrogels.

**Figure 3:**
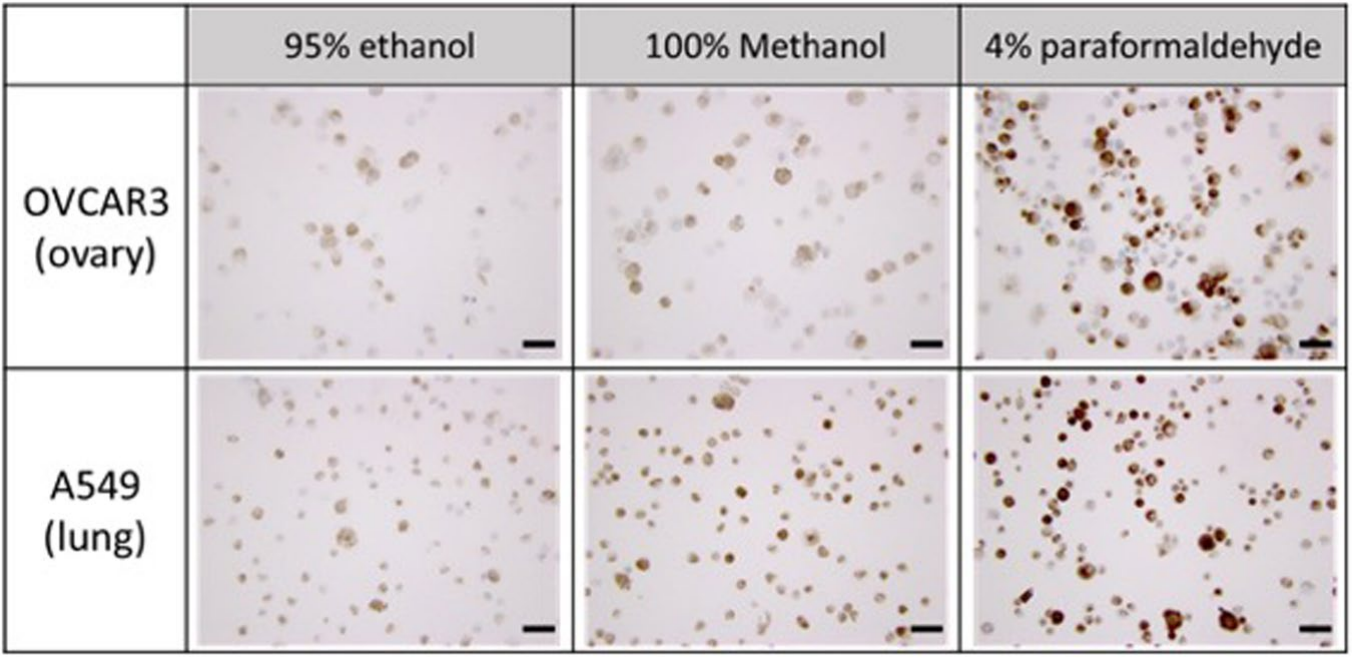
The immunocytochemical staining of cytokeratin for ovarian and lung cancer cells (OVCAR3, A549) fixed with 3 different fixative solutions (Scale bar = 50 μm).

For the cells on the slides, we considered both qualitative immunocytochemical staining results and quantitative total fixed cells on slide to choose the optimal solution. As shown in Fig. 3 (up), the slide, containing OVCAR3 and fixed with 4% paraformaldehyde, showed superior staining results after immunocytochemical staining of cytokeratin. We can see the similar results from the study with A549 (Fig. 3 (down)). Moreover, in the quantitative study measuring the total cell number after fixation, we found that almost all spiked cells (99.79–115.67%) were remained on slide after fixation with 4% paraformaldehyde (Table S1).

For the hydrogel bead, we examined both morphology of hydrogel and cell concentration in hydrogel bead after fixation. As shown in Fig. S1 (up), all beads dipped out from the three different solutions remained intact morphologically. However, the relative fluorescence in hydrogel bead showed significant difference between ethanol and others (Fig. S1 (down)). From these results using both cell slides and hydrogel beads, we conclude that 4% paraformaldehyde is the optimal fixative solution for cell block assay, and 4% paraformaldehyde was applied to both hydrogel cell blocks containing cancer cells and cytospinned slides using cancer patient blood samples.

Other steps, such as dehydration, clearing, impregnation, embedding and section cutting, were followed by conventional procedures for IHC, but incubation time and handling method were optimized for this study using hydrogel beads.

### Immunocytochemical expression of candidate markers in cell blocks containing lung, ovarian and renal cell carcinoma cell lines

After the formation of cell blocks (Fig. S2) by the help of hydrogel beads containing each cell line, the cell block was gently sectioned. The sections were placed onto the clean glass slides and immunocytochemically stained with commonly used markers for tissue blocks to identify cancer. We firstly examined the overall cell morphology and the nuclear-cytoplasmic ratio (NC ratio) using haematoxylin and eosin (H&E) stain. Secondly, we evaluated the tumor marker expressions for lung, ovarian and renal cell carcinoma.

**Figure 4:**
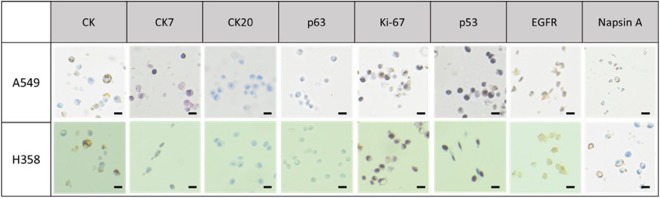
The immunocytochemical stain in lung cancer cell lines cell block (Scale bar = 20 μm).

**Figure 5:**
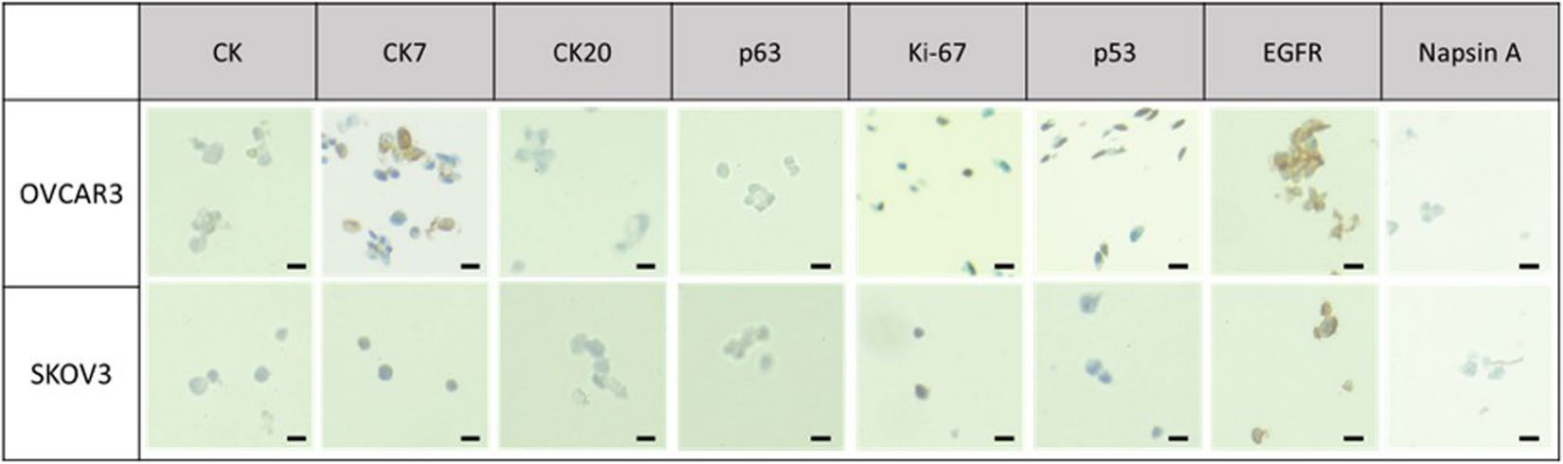
The immunocytochemical stain in ovarian cancer cell lines cell block (Scale bar = 20 μm).

For the cell blocks containing lung and ovarian cancer cells, eight different antibodies were used: cytokeratin (CK: AE1/AE3), CK7, CK20, p63, Napsin A, p53, ki-67, and EGFR. The expression of each marker for lung and ovarian cancer cells were shown in Figs 4 and 5, respectively. Three different cytokeratin were used for testing epithelial properties of cancerous tissue or cells. It is often used in combination of CK7 and CK20 to distinguish different types of glandular tumor^35^. CK7 is used to identify the origin of epithelial cells and it is expressed in various tumors such as adenocarcinoma of lung, ovary, breast, endometrium, and urothelial carcinoma of urinary bladder^35^. CK20 is usually used for identification of colorectal adenocarcinoma and urothelial carcinomas, while it is not expressed in lung, prostate, and non-mucinous ovarian cancers, including OVCAR-3 and SKOV-3 cells, which we use. As shown in Figs 4 and 5, we verified the positive expression of CK and CK7 while no significant signal was found in CK20 antibody for both lung and ovarian cancer cells. Ki-67 and p53 were frequently used as tumor marker associated with poor survival of cancer and have been usually indicated in cell proliferation and p53 mutation, respectively^36,37^. As shown in Table 1, we confirmed that Ki-67 level of lung cancer cell block for A549 and H358 shows the 70% and 80% positivity, respectively. Furthermore, we also verified that p53 expression of lung cancer (A549, H358) was significant, but is non-expressed or expressed low for ovarian cancer (SKOV-3 and OVCAR-3), which is in accordance with previous works^38,39^. In our case, EGFR mutation was found more significantly on A549 compared to that of H358. Napsin A, which was expressed only in the lung cancer cell lines, is mainly used for diagnosis of lung adenocarcinoma when compared to TTF-1 because of more sensitive and specific marker^40^. Napsin A is expressed in approximately 75% to 80% of lung adenocarcinoma, in case of utility in the identification of primary and metastatic lung adenocarcinoma among cytologically poorly differentiated carcinomas.

**Table 1:** Immunocytochemical stain in lung and ovarian cancer cell line cell block.

Marker	Lung		Ovary	
	H358	A549	OVCAR-3	SKOV-3
CK (AE1/AE3)	(++)	(++)	(+)	(+)
CK7	(+)	(+)	(+)	(+)
CK20	(−)	(−)	(−)	(−)
p53*	70%	70%	60%	(−)
Ki-67*	80%	70%	70%	70%
EGFR	(++)	(++)	(++)	(++)
P63	(−)	(−)	(−)	(−)
Napsin A	(+)	(+)	(−)	(−)

^*^Positive rate in cancer cells.

(+) focal or partial expression.

(++) diffuse expression.

**Figure 6:**
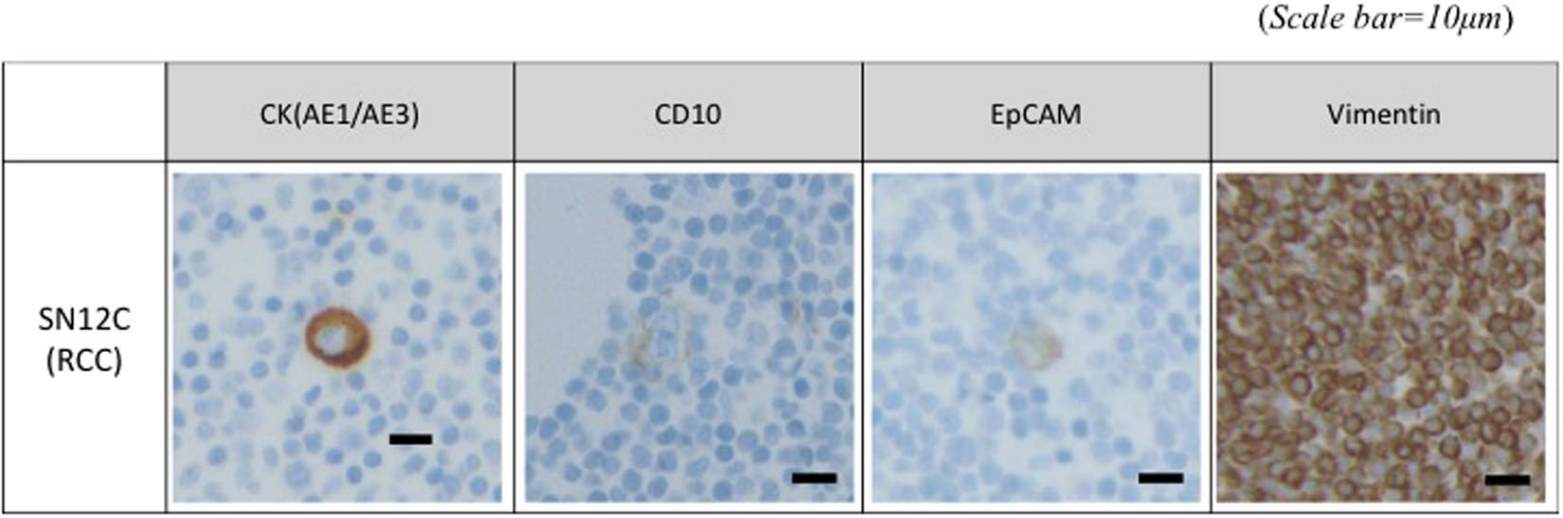
The immunocytochemical stain in RCC cell line (SN12C) cell block (Scale bar = 10 μm).

For the cell blocks containing RCC cell lines, eight different antibodies were applied and those include EpCAM, CK (AE1/AE3), CK8, EMA, CD10, CA IX, RCC, and vimentin. These eight antibodies are most widely used immunomarkers for confirmation of RCC in biopsied cancer tissue^41^. The result after immunocytochemical staining for RCC was summarized in Table 2. Among them, SN12C cell block highly expressed the four markers, EpCAM, CK (AE1/AE3), CD10, and vimentin. The 769-P cell block expressed three markers predominantly: CK (AE1/AE3), CD10, and vimentin. CK (AE1/AE3), which is called pankeratin, multi-cytokeratin, or pan cytokeratin, respectively, exhibits broad reactivity with two families of cytokeratin, acidic and basic. This cytokeratin covers CK1-8, 10, 14–16 and 19, but not cover CK17 or CK18. We confirmed that these two cell blocks containing each cell were significantly positive to CK (AE1/AE3). In addition, EpCAM, which is widely used as a marker for epithelial CTCs, was also weakly expressed on RCC cell lines. The CD10 is used to distinguish histologic type of RCC with most common ‘clear cell RCC’ from ‘chromophobe RCC’ or ‘oncocytoma’. The two RCC cell lines show positive to CD10. Vimentin was positive for the two cell lines, but it cannot be applicable for detection of CTC because leukocyte was also diffusely positive for vimentin, resulting to poor discrimination of CTC from leukocytes. Based on this result, CK (AE1/AE3), CD10, and EpCAM were chosen as the potential immunomarkers for CTC-based diagnosing for RCC (Fig. 6), and these markers were applied to clinical samples from patient with RCC.

**Table 2:** Immunocytochemical stain in RCC cell line (SN12C and 769-P) cell block with WBC.

Marker	SN12C	769-P	Leukocyte
EpCAM	(+)	(−)	(−)
CK (AE1/AE3)	(++)	(+)	(−)
LMW CK (CK8)^a^	(−)	(−)	(−)
EMA	(−)	(−)	(+/−)
CD10	(+)	(+)	(+/−)
CA IX	(−)	(−)	(−)
RCC	(−)	(−)	(−)
Vimentin	NA^b^*	NA^b^	Diffuse (+)

^*a^low molecular weight Cytokeratin (Cytokeratin 8).

^b^Not applicable: due to diffuse positivity in cancer cells as well as WBCs.

+/− occasionally positive for B lymphocytes.

+ Focal or partial positivity.

++ Diffuse positivity.

### CTC filtration from renal cell carcinoma patients’ blood samples

We extended our study using 48 clinical samples from patients with RCC. The 0.5 ml to 3.0 ml of blood samples were processed by tapered-slit filter and CTCs were successfully isolated and verified by either immunocytochemical staining or immunofluorescent staining. We verified the 27.1% (13 of 48) of CTC positivity among 48 clinical samples, and their CTC count ranged 0 to 6 per milliliter. The isolated and released cells from the microfilter were gently cytospinned on three slide glasses then followed by immunocytochemical staining with three candidate markers which we chose, CK(AE1/AE3), EpCAM, and CD10. Unlike the cancer cell block required for 8 candidate marker evaluation, clinical samples were cytospinned on only 3 slides to minimize the time and effort during hydrogel-block formation.

### Immunocytochemical expression profiles of CTCs

For the cytopathological study, positive expression was defined as the unequivocal brownish staining in the cytoplasm and/or cell membrane. Cytological criteria for tumor cells were as follow: large cell size (1.5 times larger than white blood cells), large nuclear size with high nuclear/cytoplasmic ratio, irregular nuclear membrane, and presence of cytoplasm^42^. The presence of tumor cells regardless of tumor-associated antibody expression was considered as CTC, and the total number of CTCs in entire three slides (CK, EpCAM and CD10) was counted. All slides were examined by one pathologist (Chang, H.J.).

**Figure 7:**
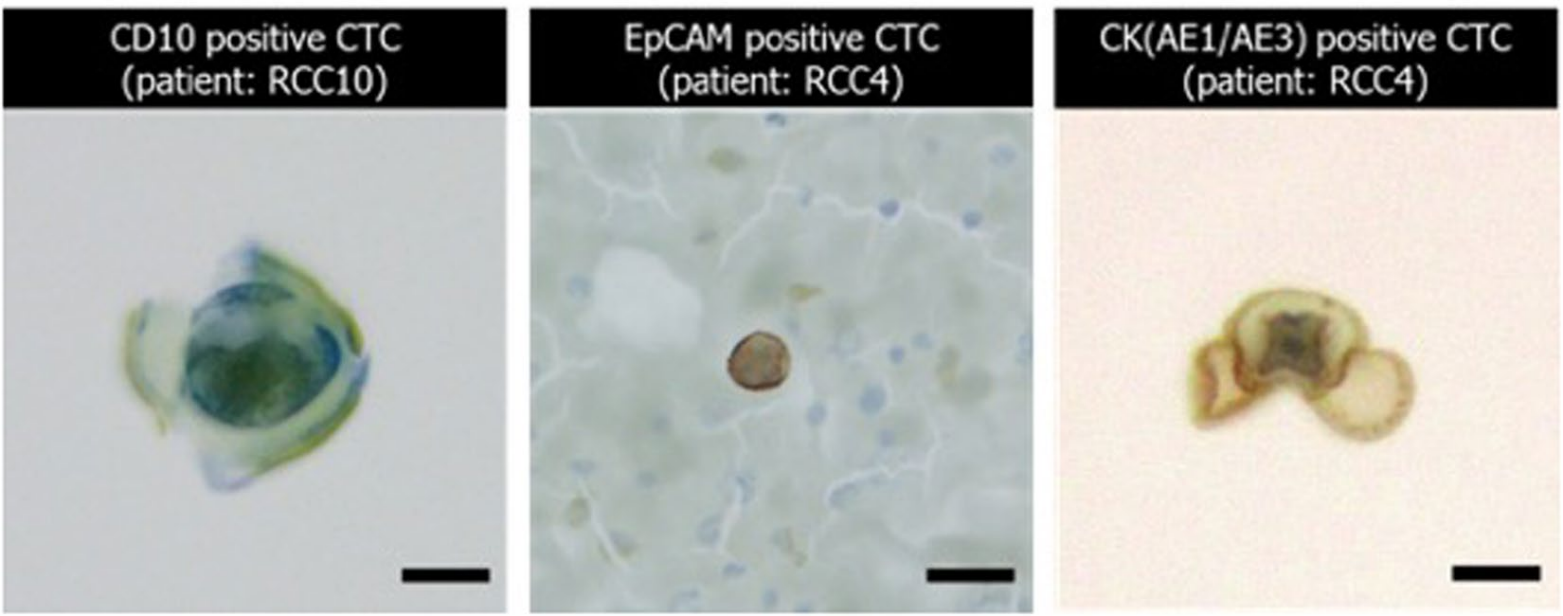
The representative images of the isolated circulating tumor cells from the patients with renal cell carcinoma by using multi-physical CTC isolation method (Scale bar = 20 μm).

CTCs were detected in 13 cases, and all isolated CTCs were morphologically intact. The overall results and descriptions of the CTC-positive cases were summarized in Table 3. The CTCs in 13 cases were CK-positive in 3 cases and/or EpCAM-positive in 5 cases (CK and EpCAM positive in 2 cases), and CD10-positive in 1 case. In the remaining 4 cases, cytologically malignant CTCs were identified in CK (3 cases) or CD10 (1 case) immunostained slides, but the CTCs were CK- or CD10-negative. For some cases, cell cluster which is EpCAM positive but non-epithelial by morphology was found (Fig. S3). We did not count them as CTCs but carefully morphological confirmation is necessary for reduce false-positive counts. The representative images of the isolated and immunocytochemically stained circulating tumor cells are shown in Fig. 7. The morphology of CTCs and their size were varying and highly irregular. Its irregularity and characteristic morphology have been confirmed from previous studies^30,31^.

**Table 3:** The description of the 13 circulating tumor cell positive cases identified by immunocytochemical (ICC) staining, and its comparison with immunofluorescence (IF) staining results.

Sample ID		Sample description				IHC		IF	
KAIST ID	SMC ID	Sex	Stage (TNM)	Metastasis	Sample volume (ml)	CTC number	Marker positivity	CTC Number (/5 ml)	EpCAM positivity
RCC1	RCC-013	M	cT3N1M0	Lymph node	3	1	CK+	0	N/A
RCC2	RCC-014	M	cT3N0M0	—	1	1	EpCAM+	0	N/A
RCC3	RCC-016	M	c T3N0M0	—	0.5	1	CK+	4	Negative
RCC4	RCC-020	F	cT4N0M1	Rt.Adrenal,lung	1	5	CD10−	0	N/A
RCC4-2	RCC-020-3	F	cT4N0M1	Rt.Adrenal,lung,lympha	3	1	EpCAM+	2	Positive
RCC5	RCC-025-2	M	cT3aN0M0	—	1	3	CK+/EpCAM+	1	Positive
RCC6	RCC-030-3	F	cT3aN1M1	pelvis, right hepatic lobe, lymphadenopathy	1	1	EpCAM+	2	Positive
RCC7	RCC-039-1	M	cT3aN0M0	—	2	1	CK−	1	Positive
RCC8	RCC-043-3	M	cT3bN0M0	—	0.5	3	CK−	7	Negative
RCC9	RCC-046-2	F	cT1bN0M1	Several nodule, left lung, R/O Bladder	0.5	3	CD10−	1	Positive
RCC10	RCC-047-3	F	cT3aN0M1	lung meta	3	1	CD10+	1	Positive
RCC11	RCC-048-1	M	c T4aN1M0		3	4	EpCAM+	0	N/A
RCC12	RCC-049-2	M	cT3N0M0		3	1	CK−	0	N/A
RCC13	RCC-029-4	M	cT4N0M1	Pulmonary	3	0	EpCAM+ non-epithelial	0	N/A

### Comparison of result identified by ICC and IF

**Figure 8:**
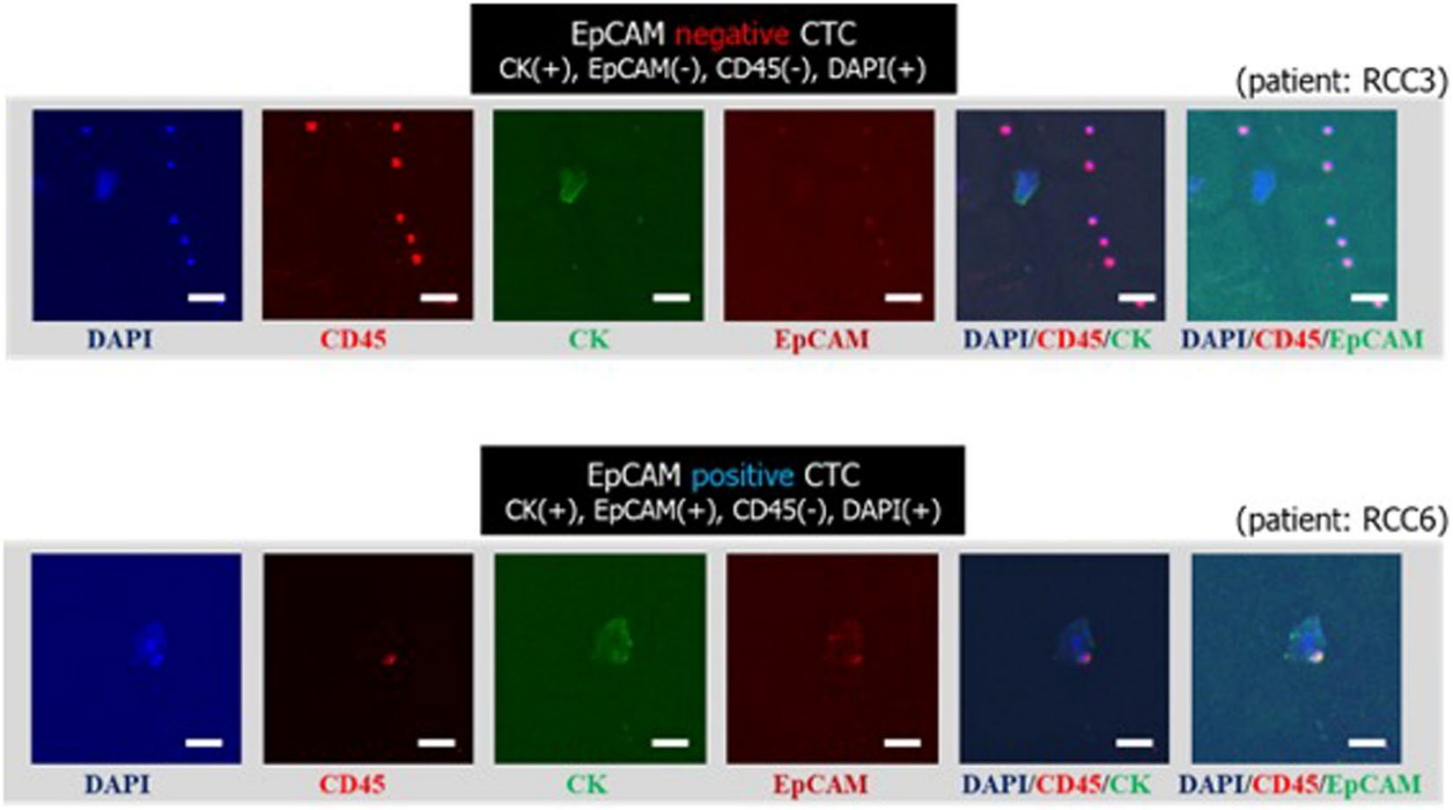
The immunofluorescent images of the isolated circulating tumor cells by the present multi-physical CTC isolation method (Scale bar: 20 μm).

Of the 13 cases showing CTC positivity by immunocytochemical staining, 8 cases (61.5%) also show CTC positivity by immunofluorescence staining and their CTC count ranged 0 to 7 per 5 milliliters. We counted the CTCs as following detection criteria, CK+/CD45−/DAPI+/EpCAM(+/−) with high nuclear-cytoplasmic ratio compared to blood cells. Among 8 cases, 6 cases have at least one EpCAM positive cell, and this result matched with 3 cases whose CTC show EpCAM expression by ICC. The representative images of the isolated and immunofluorescently stained circulating tumor cells are shown in Fig. 8. For some patients, the undefined CTC-like cells were discovered (Fig. S4). Depending on criteria for immunostaining, those cells which are CD45-positive are considered as non-CTC, however, cell with similar morphology was also found in immunocytochemical staining. Further confirmation and studies are demanding for those undefined cells.

## Discussion

Renal cell carcinoma (RCC) is one of the least studied cancers in terms of brand new diagnosis method and prognosis method. Despite the recent advances in diagnostic tools and biochemical researches, a few molecular markers for RCC have been identified to predict the response from specific therapies. Previous studies have shown that the cellular markers can be a solution to evaluate aggressive forms, in spite of the fact that clinical relevance still not fully validated^43–45^. Recently, circulating tumor cells (CTCs) have been considered as a promising marker to get the information about tumor status and metastatic potential for several cancers. Thanks to their clinical importance in diagnosis, few attempts to study RCC using CTCs have been conducted but still fell short of real clinical use. In addition, the CellSearch system depending on epithelial cell adhesion molecule (EpCAM) antibody can be limited for RCC patients due to its semi-epithelial characteristics. RCC is classified into several histologic types including clear cell, papillary, chromophobe types^46^. Clear cell RCC, the most common type of RCC show differentiation to renal tubular epithelium histologically, but unlike normal tubular epithelium, the clear cell RCCs express vimentin and CK^47^, and this feature suggest stem cell differentiation or EMT in RCC, resulting in major limitation for CTC detection in RCC patients by current EpCAM-dependent CTC analysis method.

In order to use CTC in RCC diagnosis, two problems have to be solved; the immunomarkers capable to define RCC and the non-biased CTC isolation methods regardless of expression of surface protein such as EpCAM are necessary. From the previous preliminary study using the present non-biased CTC filtration method, we successfully isolated the 87.1% of RCC cell line, SN12C spiked into non-processed blood at the high throughput condition. Although there are few reports that small sized-CTCs are in blood and it might be missed by microfiltration method^48^, considering the semi-epithelial characteristics of RCC, microfiltration method based on the size and deformability of cells still might be an appropriate approach to isolate the CTCs in RCC samples. At the finding candidate markers for RCC, the vimentin is highly expressed in cell lines, SN12C or 769-P, as well as in leukocytes in blood. Thus, we conclude that single use of vimentin as CTC marker needs careful concern for RCC diagnosis using CTC samples. So in this study, we exclude the vimentin for immunocytochemical staining applied to CTCs isolated by the present tapered-slit filter method, to avoid false-positive results.

From the cell block study using lung and ovarian cancer cell lines, we screened several applicable immunomarkers for CTCs. To date, most of studies for CTC isolation determine the CTCs using 4 panels, DAPI, CK, CD45 and EpCAM, regardless of type of cancers. Depending on the aim of the study, specific immunomarkers for specific cancers may give the better information compared to traditional standardized markers. Thus we applied various cytokeratin antibodies including CK, CK7 and CK20. We confirmed that CK7, the maker mainly used for the diagnosis of adenocarcinoma type of lung and ovarian cancer, was significantly positive for adenocarcinoma cell lines, while showing negative for CK20. These findings suggest that immunocytochemical stain of multiple organ-specific markers may be applicable for cytopathological detection of CTCs by our methods, and also applicable for diagnosis of primary origin by CTC analysis in the patients with metastatic diseases of unknown primary origin. For instance, CK7-negativity/CK20-positivity indicates colorectal origin. Positive staining for CK7 and ER and lack of diffuse positive staining for CK20 would favor an ovarian origin^49^. For lung cancer, squamous cell carcinomas frequently revealed no immune reactivity for CK7 or CK20 but are positive for CK5/6, p63, SOX2 and p40. Lung adenocarcinoma is positive for TTF-1 which is a homeodomain-containing nuclear transcription protein of the NK2 homeobox (NKX2) gene family, and it plays a crucial role in organogenesis of the thyroid gland and lung. For the determination of targeted therapy, further immunohistochemical stain for monoclonal antibodies such as EGFR. EGFR mutation is essential for deciding the appropriate chemotherapy, such as erlotinib (Tarceva) or gefitinib (Iressa) for lung cancer. Our study has several limitations in terms of preliminary study for clinical validation with limited number of cases, and the discordance of CTC numbers in RCC cases detected by two different methods (ICC vs IF) (Table 3). Differences in CTC numbers could be due to the acquisition of cellular image regardless of antibody expression in immunocytochemical staining, different amount of blood samples (1–3 ml in IHC analysis vs 5 ml in IF analysis), or rarity of CTC within blood resulting in occasional capture of CTCs. From the previous study of CTC block, we found that certain number of CTCs critically affect to successful construction of cell block^42^. We recovered almost 91.2% of spiked cells in cell block (data not shown), however, the CTC recovery rate diminished as CTC number decreased, and the number of CTC per one paraffin section was one or less when ratio of CTC to WBCs is under the 1: 106. Recent work in cell block study also showed that at least 1,000 spiked cells are needed for cell recovery after IHC procedure^32^. The high purity performance in the present device might lower the detection rate of CTCs in cell block. Similar to paraffin block, the slide after cytospinning might need certain number of cells for cytopathological study.

In spite of the limitations, our study results demonstrate the possibility of cytopathological analysis for CTC, complementary to current CTC detection systems, and the possibility of its clinical application for diagnosis. Further clinical validation for large-size sample set is remained for future work.

In conclusion, our comprehensive study including immuno-markers screening and their applicability test with clinical samples demonstrate the potential for clinical utility of the present device and hydrogel-based cell block in CTC isolation and identification.

## Methods

### Ethics statement

The experimental protocols were approved by both Samsung Medical Center (SMC) Institutional Review Board (IRB) and Korea Advanced Institute of Science and Technology (KAIST) Institutional Review Board (IRB). Informed consent was obtained from all participants. All experiments were performed in accordance with the approved guidelines and regulations.

### Cell line and culture

Human kidney cell lines, SN12C and 769-P, human lung cancer cell lines, A549 and H358, and human ovary cancer cell lines, OVCAR3 and SKOV3, were purchased from American Type Culture Collection (ATCC; Manassas, VA, USA). Cells were cultured in RPMI 1640 media with 1% (v/v) penicillin (Invtirogen Corporation, CA) and 10% (v/v) fetal bovine serum (FBS; Gibco). Cells were maintained inside the incubator (SANYO, MCO18AIC) that supplies humidified condition at 37 °C with 5% of CO2. After two days of culture, cells were trypsinized and ready to make cell blocks which contain each cell at the cell concentration of 105 cells/block. The fluorescence labeled cancer cells were additionally prepared for stability test in 7 different chemicals for IHC and finding the optimal fixative solution. For the labeling, the prepared cells were further stained with CellTracker (Thermo Fisher Scientific, Waltham) green (CMFDA) with respect to the manufacturer’s instructions.

### Blood sample preparations

The 48 blood samples from patients with renal cell carcinoma were recruited from Samsung Medical Center (SMC) according to the protocol approved by IRB. Each blood sample (10mls each) was collected in BD Vacutainer tube, transported, and used within 6 hours after sampling. Non-treated patient whole blood samples were diluted to dilution ratio of 1:2 (Blood:PBS) and fixed amounts of blood were used for 3 different experiments, pathological identification, immunofluorescent assay, and RT-PCR molecular assay. The bloods of healthy volunteers were collected under the protocol approved by IRB in KAIST.

### Preparation of the cell-laden hydrogel beads

The preparation of the cell-laden hydrogels was performed according to the previously reported literatures with a minor modification^50–52^. Briefly, sodium alginate was dissolved in deionized water at a concentration of 4% (w/v), under constant stirring at 85 °C. After the aggregate was completely dissolved, the temperature of the alginate mixture was gradually lowered to RT. Then the mixture was transferred to sterile container and kept in CO2 incubator, in order to minimize cell damage. Separately, the cultured renal cancer cells, SN12C and 769 P, were collected, centrifuged, and re-suspended in fresh medium, at the appropriate cell density. Thereafter, these re-suspended cell solutions were gently mixed to the prepared homogenized alginate solution at 1:1 volume ratio, thus the final concentration of alginate is estimated to be around 2% (w/v). The cell-alginate mixture, which had been repetitively blended, was loaded into 100 mM calcium chloride solution drop by drop using volume pipette, followed by 15 minutes of further incubation for curing with constant stirring. The formed cell-laden hydrogel beads were carefully washed several times by deionized water and stored in PBS until further use.

### Preparation of Paraffin-embedded Cell Blocks Using Cell-laden Hydrogels

The cell-laden hydrogel beads were applied to the commonly used procedure for paraffin tissue blocks. First, the beads were fixed in 4% formalin, followed by dehydration process with 70% ethanol, 80% ethanol, 90% ethanol, 95% ethanol, and 100% ethanol. Each process was achieved by immersing beads into the prepared solvent for 3 min without repetition: these conditions were determined by considering differences between tissues and cell-laden hydrogel beads. Also, the beads were carefully handled using the spoon-shaped lab spatula, through the entire process. After that, the beads were incubated with xylene solution for 2 times and dipped in 20 minutes; xylene was utilized as an intermediate solvent, to infiltrate the dehydrated beads to paraffin wax. Then, these prepared beads were taken out of the xylene solution and placed in the embedding cassettes. After closing lid, the cassettes were embedded into the molten paraffin wax. Because the paraffin wax is liquefied at about 55~60 °C, it can be solidified with the embedded beads when the temperate lowered to 4 °C. The prepared cell blocks were kept in shade until further processing for Immunocytochemical staining (ICC).

### Optimization of protocols for ICC staining

There are three kinds of solutions to fix cells for immunocytochemical staining. 95% ethanol, methanol, and 4% paraformaldehyde are most favored fixative solutions for IHC. The fixation method is decided depending on the sample and their components. In order to find the optimal fixation method for best immunocytochemical staining results, we spiked 4,000-7,000 cells on slide glass, and then fixed the cells using three different fixative solutions for 30 min. These slides followed by cytokeratin staining and total cell number and staining results were examined to decide the optimal fixative solution for this study. The total cell number was automatically counted by Apero Image Scope software program (Aperio Technologies, Vista, CA, USA) after scanning of the slides using as Aperio ScanScope (Aperio Technologies) In order to validate immunocytochemical application of RCC tumor markers to RCC cell lines of SN12C and 769-P, two cell blocks were also constructed according to previously reported method^42^. To identify useful markers, healthy donor blood was spiked with the SN12C and 769-P at a ratio of 1:104. Cell blocks containing RCC cells and leukocytes were cut and sections were immunostained with antibodies to CK, EpCAM, CK8, EMA, CD10, CAIX, RCC, and vimentin. Details of the antibody information are shown in supplementary table.

### Fluorescence verification of cell-laden hydrogel beads using ***In-Vivo*** Imaging system

In order to examine the cell existence and stability in several solutions for IHC, we made the hydrogel beads containing fluorescence-labeled cancer cells, followed by dipping in and out from the solutions. The recovered cell-laden hydrogel beads examined by Xenogen *In-Vivo* Imaging System (PerkinElmer, Waltham, MA) to obtain whole-body fluorescence images in non-destructive way. The FITC signals from the embedded cells were detected at an excitation wavelength of 490 nm and an emission wavelength of 525 nm (GFP: 395 nm/ 509 nm). Fluorescence images were obtained during 1 second of exposure time (f/stop = 2), and bright-field photographs were also taken simultaneously. Those images of hydrogel beads were merged and analyzed using Living Image 4.52 software (Caliper Life Sciences).

### Immunocytochemical staining and profiling of antibody expression of cell blocks

Hydrogel-based cell block specimens were fixed with buffered formalin and embedded in paraffin. Sections of 4-5 μm thickness were placed on glass slides, heated at 60 °C for 30 min, and then deparaffinized with xylene and ethanol. Each slide were automatically immunostained by using the BenchMark XT Slide Preparation System (Ventana Medical Systems, Inc., Tucson, AZ, USA), with candidate antibodies. In brief, specimens mounted on glass slides were immersed in heated antigen retrieval solution for 60 min and allowed to cool for 20 min at room temperature in order to antigen retrieval. After the inactivation of endogenous peroxidase, primary antibody was reacted for 1 hour. After reaction, cells were washed in wash buffer (DAKO)-three times and were reacted with EnVisio FLEX/HRP (DAKO) for 40 min. Afterwards, the samples were washed in wash buffer (DAKO)-three times and incubated with DAB reagent (3,3-diaminobenzidine tetrahydrochloride) (DAKO) for 10 min. Samples were washed with deionized water (three times, 3 min respectively) and were stained with modified Mayer’s hematoxylin (Thermo scientific, MA, USA) for 3 min and were washed with deionized water (three times, 3 min respectively). The stained slide was examined microscopically. The slides were mounted using neo-mount (Merck Millipore, Massachusetts, USA) and assessed using Axiovert25 microcopy (Carl Zeiss, Jena, Germany). Positive expression was defined as the unequivocal brownish staining in the cytoplasm and/or cell membrane.

### Label-free circulating tumor cell isolation

The label-free physical CTC filtration method used here was proposed recently^30,53^. This method uses tapered-slit filter having the diameter of 20 mm and containing 625x625 (=165,625) tapered-slits at the slit density of 52,727/cm^2^, thus facilitating high throughput isolation at the flow rate up to 60 ml/h. Each oval-shaped tapered-slits designed with wider inlet and the narrower outlet in depth facilitate, thus achieving not only viable isolation while maintaining their original shapes, but also selective CTC isolation from other blood cells having same size but different ability to deform at slits. Their fabrication procedure and basic operational methods are identical to our previous published works^30,53^, however, overall CTC filtration and retrieval conditions were modified and optimized for this work, which is mainly focused on pathological study of isolated cancer cells from human blood samples with cancer. The donated blood samples were passed through the device at the flow rate of 30 ml/h, and captured cells were gently released by applying reverse flow of PBS solution (10 ml). Each released CTC sample was cytospinned on slide glass and examined by immunofluorescence or immunocytochemical staining.

### Immunofluorescent staining of CTCs

Cells captured by the microfilter device were analyzed by either immunofluorescence staining or immunocytochemical staining. After the captured cells were cytospinned on the slide glass, cells were fixed with 4% paraformaldehyde solution for 30 min. The solution was gently aspirated and cells at the slide glass were immediately rinsed with PBS solution, 3 times for 5 min each. Next, permeabilizing and blocking processes were sequentially done for 30 min each, using Triton X-100 (Fisher Scientific) and PBS solution containing 3% bovine serum albumin (BSA, Sigma Chemicals), respectively. Finally, the cells were labelled using prepared immunofluorescence staining solution containing FITC-conjugated anti-cytokeratin (15:1000 diluted in PBS, Cat.# 130-080-101, MACS Miltenyi Biotec.), PE-conjugated mouse anti-human CD45 (30:1000 diluted in PBS, Cat.# 555483, BD Pharmingen), Alexa fluor 647- conjugated anti-human EpCAM (15:1000 diluted in PBS, Anti-CD326, Cat.#324212, BioLegend), and DAPI (10:1000 diluted in PBS D1306, Life Technologies) for 1 hour. Then, excess dyes were aspirated and washed 3 times with PBS solution. The fluorescence images were acquired and examined using MetaMorph software (Molecular Devices, USA). All immunofluorescent cells were examined carefully, considering both staining criteria (Cytokeratin+/DAPI+/CD45−) and morphological features, such as size, irregularity, and nucleus-to-cytoplasm ratio.

### Immunocytochemical staining of CTCs

For pathological confirmation and finding various types of CTCs, immunocytochemical staining was conducted for captured cells from blood samples donated by cancer patients with RCC independently. All captured and released cells from the microfilter were gently cytospinned on slide glass and fixed with 4% paraformaldehyde in 15 minutes. After fixation step, each slide were automatically immunostained by using the BenchMark XT Slide Preparation System (Ventana Medical Systems, Inc., Tucson, AZ, USA), with antibodies to EpCAM (clone VU-1D9, 1:2,000, Calbiochem, San Diego, CA, USA), CK (clone AE1/AE3, 1:100, Dako Cytomation, Glostrup, Denmark), and CD10 (clone 56C6, 1:400, Novocastra Leica Biosystems, NewCastle, UK). Positive expression was defined as the unequivocal brownish staining in the cytoplasm and/or cell membrane. Cytologic criteria for tumor cells were as follow: large cell size (1.5 times larger than white blood cells), large nuclear size with high nuclear-cytoplasmic ratio, irregular nuclear membrane, and presence of cytoplasm^42^. The presence of tumor cells regardless of their EpCAM, CK (AE1/AE3), or CD10 expressions was considered as CTC, and the total number of CTCs in entire slide was enumerated. All slides were examined by one pathologist (Chang, H.J.).

## Electronic supplementary material

Supplementary Information
