# Supplementary material for: Cytopathological Study of the Circulating Tumor Cells filtered from the Cancer Patients’ Blood using Hydrogel-based Cell Block Formation
Source: Sci Rep. 2018 Oct 12;8:15218. doi: 10.1038/s41598-018-33464-1 (PMC6185971; doi:10.1038/s41598-018-33464-1)
Supplement: Supplementary file 1 — Supplementary Information [file 41598_2018_33464_MOESM1_ESM.docx]

**Supplementary Material**

**Cytopathological Study of the Circulating Tumor Cells filtered from the Cancer Patients’ Blood using Hydrogel-based Cell Block Formation**

**Yoon-Tae Kang**^1^**, Young Jun Kim**^1^**, Tae Hee Lee**^1^**, Young-Ho Cho**^1,*^**,** **Hee Jin Chang**^2,*^**,** **Hyun-Moo Lee**^3^

^1^ Cell Bench Research Center, Korea Advanced Institute of Science and Technology, 291 Daehak-ro, Yuseong-gu, Daejeon 34141, Republic of Korea

^2^ Research Institute and Hospital, National Cancer Center, 323323 Ilsan-ro, Ilsandong-gu, Goyang-si Gyeonggi-do, 10408, Republic of Korea

^3^ Samsung Medical Center, 81 Irwon-ro, Gangnam-gu, Seoul, 06351, Republic of Korea

* Correspondence to: Young-Ho Cho (nanosys@kaist.ac.kr) and Hee Jin Chang (heejincmd@ncc.re.kr)

**CONTENTS**

**Supplementary tables**…………………………………………………………….…….….……**2**

**Supplementary figures**……………………………...…………………………….…….….…...**3**

**S1. The antibody information**……………………….……………………………………….…**7**

**S2. Fluorescence In Situ Hybridization (FISH) analysis in cell block** ……….………………**8**

**Reference**…………………………………………………………………………………………**9**

**Table S1**. The spiked and retrieved cell number after fixation of cell containing slide with 4% paraformaldehyde.

|  | **Spiked cell number** | **Retrieved cell number**  **(%)*** |
| --- | --- | --- |
| A549  (Lung) | Approximately  6770 | 7831  (115.67%) |
| OVCAR3  (Ovary) | Approximately  4390 | 4381  (99.79%) |

*Automatic counting by image analyzer program (Aperio Image Scope Software)

**
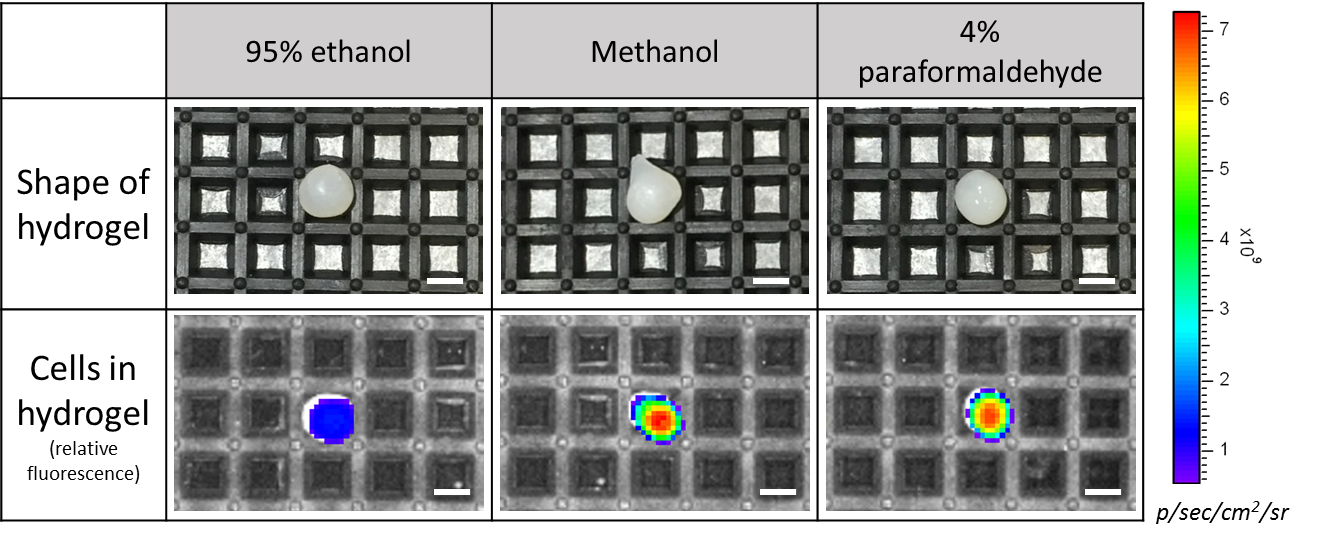
Figure S1**. The immunocytochemical staining of cytokeratin for ovarian and lung cancer cells (OVCAR3, A549) fixed with 3 different fixative solutions (*Scale bar=3mm*)


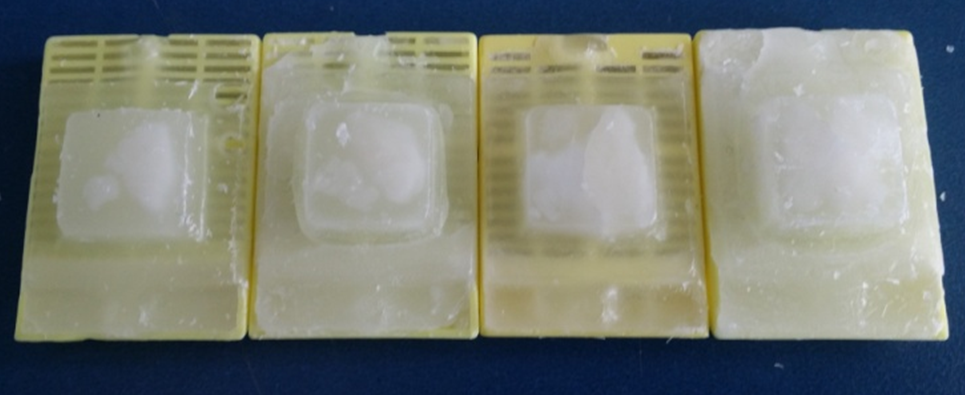


**Figure S2**. The fabricated cell block containing cancer cell-laden hydrogel bead for immunocytochemical staining of circulating tumor cells


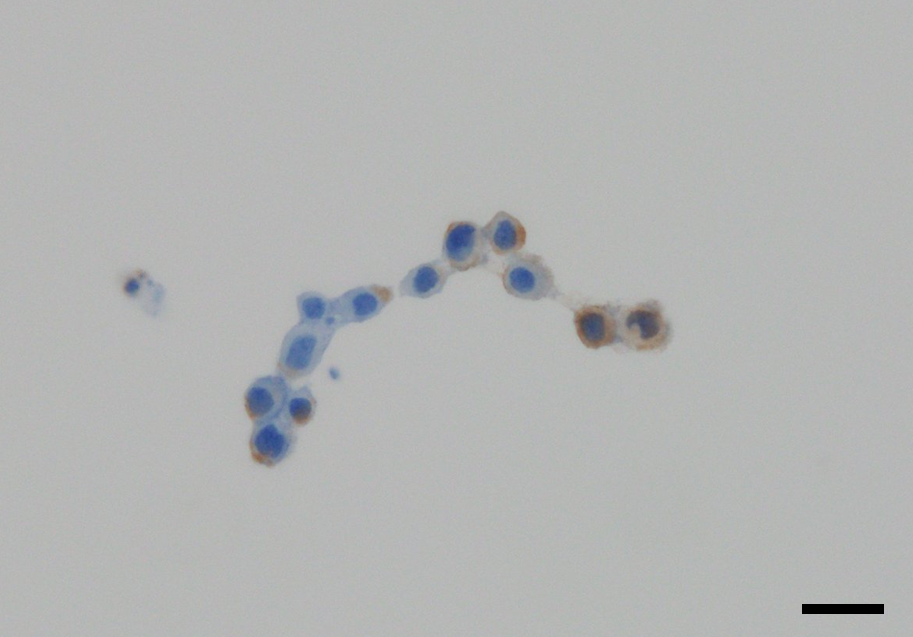


**Figure S3**. The EpCAM positive non-neoplastic cells of renal cell carcinoma patient (Lt.RCC13), captured by multi-physical CTC isolation method (*Scale bar=20μm*)


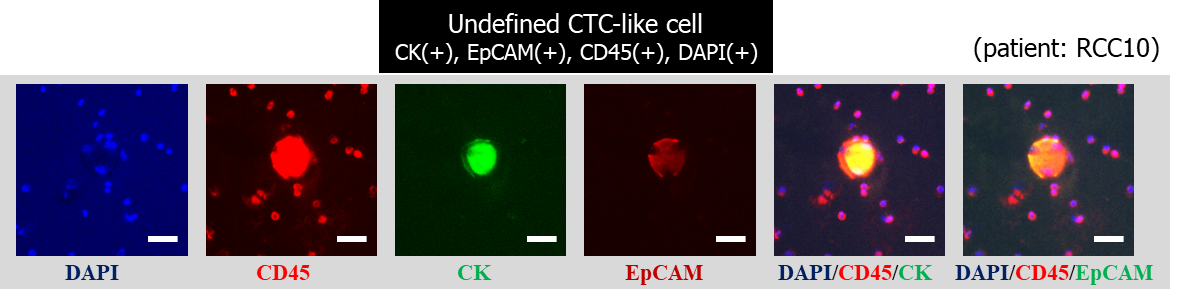
**Figure S4**. The immunofluorescent images of the captured circulating tumor cells by the present microfilter (*Scale bar: 20μm)*

**S1. The antibody information**

|  | Clone | Dilution | Corporation | Country | |
| --- | --- | --- | --- | --- | --- |
| CK | AE1/AE3 | 1:300 | DAKO | Glostrup | Denmark |
| CK7 | OV-TL 12/30 | 1:300 | DAKO | Glostrup | Denmark |
| CK20 | M7019 | 1:300 | DAKO | Glostrup | Denmark |
| p63 | DAK-p63 | 1:200 | DAKO | Glostrup | Denmark |
| Ki-67 | SP6 | 1:300 | Invitrogen | CA | USA |
| p53 | DO-7 | 1:300 | DAKO | Glostrup | Denmark |
| EGFR | 18C9 | 1:100 | DAKO | Glostrup | Denmark |
| Napsin A | TMU-Ad 02 | 1:150 | Biocare medical | CA | USA |
| EpCAM | VU-1D9 | 1:2000 | Calbiochem | CA | USA |
| LMW CK (CK8) | 35βH11 | 1:400 | DAKO | Glostrup | Denmark |
| EMA | GP1.4 | 1:200 | Novocastra | Newcastle | UK |
| CD10 | 56C6 | 1:400 | Novocastra | Newcastle | UK |
| CA IX | Rabbit polyclonal | 1;200 | Novus | CO | USA |
| RCC | 66.4.C2 | 1:200 | Novocastra | Newcastle | UK |
| Vimentin | VIM 3B4 | Ready-to-use | Ventana | AZ | USA |

**S2. Fluorescence In Situ Hybridization (FISH) analysis in cell block**

For the additional verification of cancer cell block, we extended our study to Fluorescence In Situ Hybridization (FISH) analysis in peripheral blood cell block spiked with breast cancer cell line SK-BR-3 at a ratio of 1: 1000 white blood cells. We followed the general procedure of FISH analysis in tissue block [R1] with minor optimization and changes. Using the serial sections of the cell block, we performed general H&E staining for cell identification, cancer specific marker staining, and FISH analysis. From this research, we confirmed the HER2/new gene amplification in SK-BR-3 cell block with positive expression of C-erbB2 cancer marker.

**
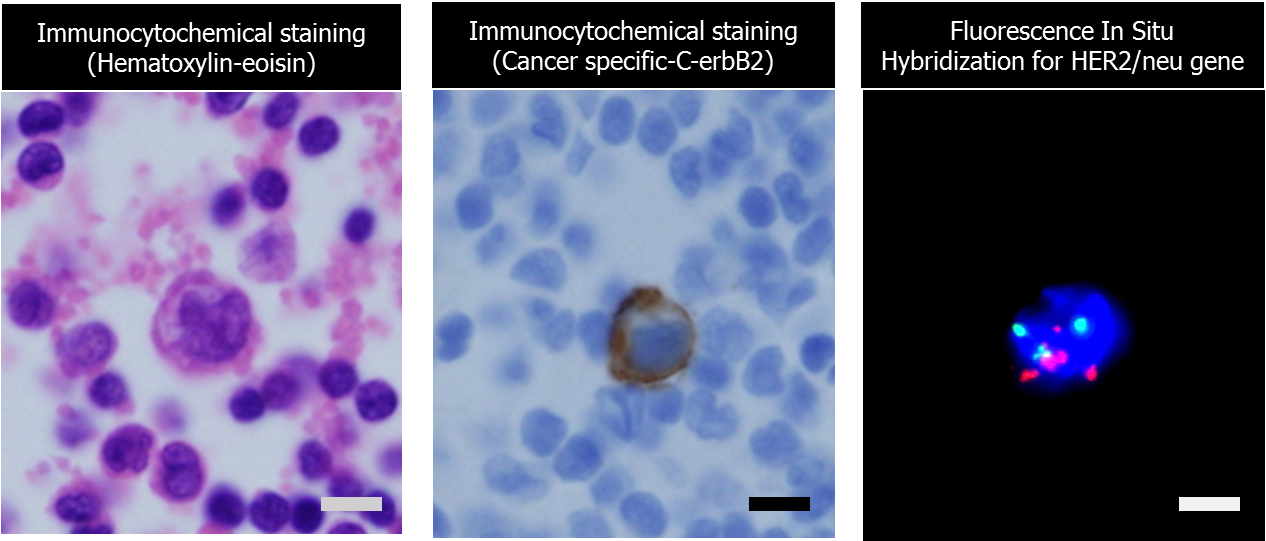
**

**Figure S5**. The immunocytochemical staining and gene amplification evaluation using SKBR3 breast cancer cell within the peripheral blood cell block (*Scale bar=10μm*).

**Reference**

[R1] Summersgill, B.M. & Shipley, J. M. Fluorescence In Situ Hybridization Analysis of Formalin Fixed Paraffin Embedded Tissues, Including Tissue Microarrays. In: Bridger J., Volpi E. (eds) Fluorescence in situ Hybridization (FISH). Methods in Molecular Biology (Methods and Protocols). Methods in Molecular Biology (Methods and Protocols) 659. Humana Press, Totowa, NJ (2010)
